# The Graphic Arts in Medicine

**Published:** 1890-02

**Authors:** 


### The Graphic Arts in Medicine.

On Friday, November 1st, a meeting was held in the large theatre of the medical school of St. George's Hospital, with Sir Prescott Hewett in the chair, to inaugurate a society for the encouragement of the pictorial and allied arts amongst past and present students of the hospital. In opening the meeting the chairman expressed his opinion of the extreme value of drawing and painting to the medical men, not only for the actual results produced, but also, if seriously followed, on account of the value of the training. He then related how his pre-medical career had been passed in a French studio, and how the training had developed his accuracy of sight, and of what great importance this had been to him in his surgical work. Referring to photography, he mentioned that its importance was becoming daily more and more recognized, both in clinical and museum work, and reminded his hearers that modern photography owed its recent great progress to the enthusiasm of amateurs. Dr. Dickinson formally moved: "That a society be formed in connection with St. George's Hospital for the purpose of encouraging sketching, painting, engraving. modeling, carving, photography, and the arts of representation in general." One of the ways in which it was proposed to attain this end was to hold a meeting of the society at least once a year, at which members should exhibit any of their productions that could be included under the above headings. The meeting closed, after the election of officers and council, with a very cordial vote of thanks to Sir Hewett for taking the chair, and the society is fortunate to have secured him as its first President. Past students of St. George's who may be desirious of joining the society are requested to send their names to Dr. Penrose, the Honorary Secretary, at the hospital.—*British Medical Journal.*

